# The immunogenicity of human-origin therapeutic antibodies are associated with V gene usage

**DOI:** 10.3389/fimmu.2023.1237754

**Published:** 2023-09-01

**Authors:** Zicheng Hu, Sivan Cohen, Steven J. Swanson

**Affiliations:** Department of BioAnalytical Sciences, Genentech Inc, South San Francisco, CA, United States

**Keywords:** immunogenicity, anti-drug antibodies (ADA), therapeutic antibodies, antibody engineering, VDJ recombination

## Abstract

Therapeutic antibodies can elicit unwanted immune responses in a subset of patients, which leads to the production of anti-drug antibodies (ADA). Some of these ADAs have been reported to effect the pharmacokinetics, efficacy and/or safety of the therapeutic antibodies. The sequence diversity of antibodies are generated by VDJ recombination and mutagenesis. While the antibody generation process can create a large candidate pool for identifying high-affinity antibodies, it also could produce sequences that are foreign to the human immune system. However, it is not clear how VDJ recombination and mutagenesis impact the clinical ADA rate of therapeutic antibodies. In this study, we identified a positive correlation between the clinical ADA rate and the number of introduced mutations in the antibody sequences. We also found that the use of rare V alleles in human-origin antibody therapeutics is associated with higher risk of immunogenicity. The results suggest that antibody engineering projects should start with frameworks that contain commonly used V alleles and prioritize antibody candidates with low number of mutations to reduce the risk of immunogenicity.

## Introduction

Antibody-based therapeutics have been developed and used to treat a variety of diseases, including cancer, autoimmune diseases, allergy, infectious disease and many more (1). As of Dec 19, 2022, 111 antibody-based therapeutics have been approved by the FDA and many new antibodies are currently in clinical development (1–3). As a type of protein-based drugs, therapeutic antibodies have been documented to induce immune reactions against the therapeutic molecules, which leads to the generation of antidrug antibodies (ADAs) (4). The ADA responses can lead to reduced pharmacokinetics and efficacy of the therapeutic antibodies (4–7). In addition, ADA can induce hypersensitivity reactions in some patients, impacting the safety profile of the drugs (8–10). Because of ADA’s potential for impact on safety and efficacy, major regulatory agencies (e.g. FDA and EMA) require the manufacturers of the therapeutic antibodies to report the immunogenicity of the drug and its clinical impact on patients (11–13).

With modern antibody engineering technology, it is now a routine practice to humanize the candidate antibodies or develop human-origin monoclonal antibodies. Compared to animal-origin antibodies and chimeric antibodies, human and humanized antibodies show significantly lower immunogenicity (14, 15). However, ADA responses are still observed in some patients who receive human or humanized antibodies. For example, data from clinical trials show that 57% of patients who received teplizumab developed ADA and 23% of patients who received golimumab developed ADA (16).

ADA can be generated by T-cell dependent or independent B cell activation pathways (17). In the T cell dependent pathway, linear epitopes that are presented by antigen presentation cells activate the CD4+ naive T cells, which differentiate into helper T cells. Following the cognate interactions with helper T cells, the B cells differentiate into plasma cells and produce ADA (18, 19). To develop high-affinity ADA or neutralizing ADA responses, the B cells presumably go through an affinity maturation process with the help and regulation of follicular helper T cells and follicular regulatory T cells at the germinal center (20). In the T cell independent pathway, the therapeutic antibodies (sometimes in an aggregated form) crosslink B cell receptors and stimulate the production of ADAs (21, 22). In both cases, non-self linear or 3D epitopes are required to activate the T and B cells.

The sequence diversity of antibodies is generated by two mechanisms, VDJ recombination and mutagenesis. For therapeutic antibodies, the mutagenesis refers to both the somatic hypermutation that happens *in vivo* and the experimentally induced mutation that happens *in vitro*. Among the diverse sequences generated, many are foreign to the human immune system and can elicit ADA responses. A previous study has shown that the unique sequences in the CDR regions of human or humanized antibodies can induce the activation of CD4+ helper T cells (14). However, it is not clear how VDJ recombination and mutagenesis specifically contribute to the immunogenicity of therapeutic antibodies.

In this study, we explored how the immunogenicity of therapeutic antibodies is associated with different aspects of VDJ recombination and mutagenesis, including V and J allele usage and the number of introduced mutations. By analyzing 93 antibody drugs, we found a positive correlation between the clinical ADA rate and the number of introduced mutations. We also found that the use of rare V alleles in human-origin antibody therapeutics is associated with a higher risk of immunogenicity. The results suggest that antibody engineering projects should start with frameworks that contain popular V alleles and prioritize antibody candidates with low number of mutations to reduce the risk of immunogenicity.

## Methods

### Data collection

The B cell reservoir sequencing data were downloaded from the Observed Antibody Spaces (OAS) as of Oct 05 2022 (23). We filtered the OAS data to include datasets that are from human samples and characterize both the light chain and heavy chain of the B cell receptors. To profile the bulk B cell reservoir, we excluded datasets that only profile certain isotypes (e.g. IgG or IgM). We also excluded samples with low numbers of reads (a total number of reads less than 2000). In total, the filtered dataset contains 3300237 reads (2091286 light chain reads and 1208951 heavy chain reads) from 199 subjects.

The amino acid level sequences of the 93 therapeutic antibodies were obtained from the IMGT monoclonal antibody database (mAb-DB) (24). The clinical ADA rate information was obtained from the immunogenicity section of FDA labels. In cases where multiple ADA rates are available from different clinical trials, the maximum rates reported by monotherapy trials were used. The ADA rate of other antibodies (antibodies withdrawn from the market or investigational antibody drugs) were obtained from published literature, previous FDA labels or the Genentech internal database. Other information about the therapeutic antibodies, including the approval status, the year of the first US approval, the first approved indication and the origin (human or humanized) was obtained from the Antibody Society website.

### Calling of V and J alleles

For the OAS dataset, the V and J alleles were identified by the OAS team based on previously published workflow and were included as part of the OAS dataset (23). Briefly, The nucleotide sequences of the B cell repertoire sequencing data were aligned to germline and translated using IgBLASTn 1.17.1 (25). The VJ germline databases for humans were created using ImMunoGeneTics (IMGT) germline sequences derived from IMGT (24). For the therapeutic antibodies, the V and J alleles were identified by aligning the amino acid sequences of therapeutic antibodies to the amino acid-level sequences of the germline V or J alleles. The amino acid-level sequences of the germline V or J alleles were obtained from the gene database (GENE-DB) of IMGT (24). The germline sequence with the smallest number of mismatches with the antibody sequence was selected. In some cases, multiple alleles could be selected due to a tie in the number of mismatches. These alleles were all considered as the matching alleles in the downstream analysis.

### Allele usage quantification

The allele usage of V and J segments were measured by dividing the number of sequences that use the V or J allele by the total number of sequences in the B cell repertoire sequencing dataset. The allele usage was first measured in the B cell repertoire of each individual. The median percentage of the V and J alleles was used to represent the allele usage in the OAS dataset. For therapeutic antibodies whose sequence only has one closest allele, the usage of the allele was used to correlate with the immunogenicity. For therapeutic antibodies whose sequence has more than one closest allele with the same number of mismatches, the percentages of the alleles were summed up.


$$V\;allele\;usage\;=median\left(\;\frac{num.\;of\;BCRs\;with\;the\;V\;allele\;in\;a\;repertoire}{Total\;num.\;of\;BCRs\;in\;the\;the\;repertoire}\right)$$

### Gene usage quantification

The gene usage of V and J segments were measured by dividing the number of sequences that use the V or J gene by the total number of sequences in the B cell repertoire sequencing dataset. The gene usage was first measured in the B cell repertoire of each individual. The median percentage of the V and J gene was used to represent the allele usage in the OAS dataset. For therapeutic antibodies whose sequence only has one closest gene, the usage of the gene was used to correlate with the immunogenicity. For therapeutic antibodies whose sequence has more than one closest gene with the same number of mismatches, the percentages of the genes were summed up.


$$V\;gene\;usage\;=median\left(\;\frac{num.\;of\;BCRs\;with\;the\;V\;gene\;in\;a\;repertoire}{Total\;num.\;of\;BCRs\;in\;the\;repertoire}\right)$$

### Gene deletion quantification

The deletion of V genes are detected using a previously published criteria (26). Briefly, V genes with percentage below 0.1% were set as candidates for deletion in the sample. A binomial test was performed to test if the percentage of the candidate gene was significantly lower than the percentage of the gene in non-deletion-candidate samples. For a given gene, candidate samples with a FDR below 0.01 were marked as deleted. The frequency of deletion was defined as the number of individuals in which a V gene was deleted divided by the total number of individuals in the OAS dataset.


$$frequency\;of\;V\;gene\;deletion\;=\;\frac{num.\;of\;individuals\;without\;the\;V\;gene\;in\;the\;genome}{Total\;num.\;of\;individuals}$$

### Allele frequency quantification

For each non-deleted-gene, an individual can either have one (homozygous) or two (heterozygous) alleles of the gene. However, because of the introduced random mutations, a sequence in B cell repertoire has a low but non-zero probability to be mapped to an allele even if the allele is not present in the genome. We reason that, despite the existence of mutations, the majority of BCR sequences will be matched to its originating alleles. For a given gene, we first calculate the abundance of the alleles by dividing the number of sequences with a certain allele by the number of sequences with the gene that the allele belongs to. The allele is set as the candidate allele if its abundance is greater than 20%. For each individual, If there is only one candidate allele, the person is annotated as homozygous. If two or more alleles are set as candidates, the individual is annotated as heterozygous of the top two alleles. The frequency of the allele is measured as the number of individuals with a certain allele divided by the total number of individuals who carry the gene in the OAS dataset.


$$\;frequency\;of\;a\;V\;allele\;=\;\frac{Num.\;of\;individuals\;that\;carries\;the\;V\;allele}{Num.\;of\;individuals\;that\;carries\;the\;V\;gene}$$

### Statistical analysis

Spearman correlation was used to measure the association between clinical ADA rate and variables of interest, including the number of mismatches, the allele usage, gene usage, gene deletion frequencies, and allele frequencies. Spearman correlation was used to measure the correlation of allele usage between different disease cohorts. T-test was used to compare the differences of mismatch numbers between human and humanized antibodies. As a rank-based correlation metrics, spearman correlation does not require data transformation to reduce the effect of outliers. However, we used log transformation to variables that are not normally distributed for data visualization purposes. For data that contain zeros, pseudo counts are added to allow log transformation.

Multivariable linear regression was used to test the association between ADA rate and V gene usage among human-origin antibodies while controlling for other factors that could impact the immunogenicity, including the class of drug target, the disease category (based on drug’s first approved indication) and the year of drug’s first approval. The V gene usage was measured as the log2(percent of sequences with light chain V * percent of sequences with heavy chain V). The purpose of the log transformation was to reduce the effect of outliers and make the data normally distributed.

R (version R-4.1.1) was used for all statistical analysis (27).

## Results

### Quantifying the usage of V and J alleles using B cell receptor repertoire sequencing data

It has been long known that the usages of different V and J genes in the B cell receptor (BCR) repertoire are not equal (28, 29). Some V and J genes are more frequently used, both in terms of the number of sequences and in terms of the number of unique clones, than other V and J genes. In addition, V and J genes are polymorphic in the human population (30, 31), which could increase the chance of immunogenicity even if the antibody is humanized or has human-origin. For example, there are 14 amino acid differences between the IGHV4-34*10 and IGHV4-34*11 alleles, a 14.4% sequence difference. An antibody that uses the IGHV4-34*10 allele could potentially be seen as a foreign antigen in patients that only carry the IGHV4-34*11 allele.

**Figure 1:**
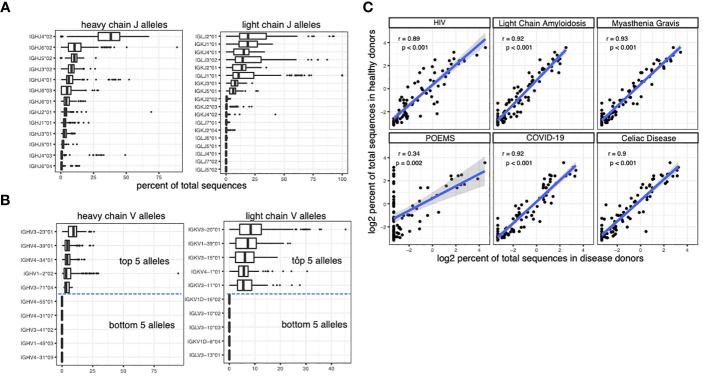
caption: Quantifying the usage of V and J alleles using BCR repertoire sequencing data. **(A)** The percent of sequences that are matched to the J alleles in the BCR repertoire sequencing dataset from OAS. Each data point in the box plots represents the percent in an individual. **(B)** The percent of sequences that are matched to the V alleles in the BCR repertoire sequencing dataset from OAS. Due to the large number of V alleles, only the top and bottom 5 alleles are plotted. **(C)** The correlation between the usage of heavy chain V alleles in healthy individuals and the usage of heavy chain V alleles in individuals with various diseases. Spearman correlation is used to measure the associations in **(C)**.

We leveraged the BCR repertoire sequencing data from the observed antibody space (OAS) database to quantify the usage of different V and J alleles in the BCR repertoires of 199 individuals (23). We identified the closest somatic V and J alleles of each sequence in the BCR sequencing data and quantified the usage of the alleles as the percent of total sequences. V and J alleles from both heavy and light chains show imbalanced usage, with some alleles used in > 10% sequences and other alleles rarely being detected (**Figures 1A, B**, see the accompanying dataset (32)).

The OAS dataset includes not only samples from healthy donors but also samples from patients with different diseases, including COVID-19, POEMS syndrome, celiac disease, HIV infection, Light Chain Amyloidosis and Myasthenia Gravis. Our analysis shows that the usage of V and J genes are largely conserved across different disease cohorts (**Figure 1C**).

### The clinical ADA rates are associated with the number of mismatches between therapeutic antibodies and the germline

We then examined the V and J alleles that are used in the therapeutic antibodies. We analyzed the sequences of 93 human or humanized antibody drugs. Among them, 50 antibodies are humanized and 43 antibodies have human-origin. 71 antibodies are commercially available and 22 antibodies are either investigational or withdrawn from the market. Bispecific antibodies, antibody-drug conjugates and chimeric antibodies are excluded from the current analysis.

**Figure 2:**
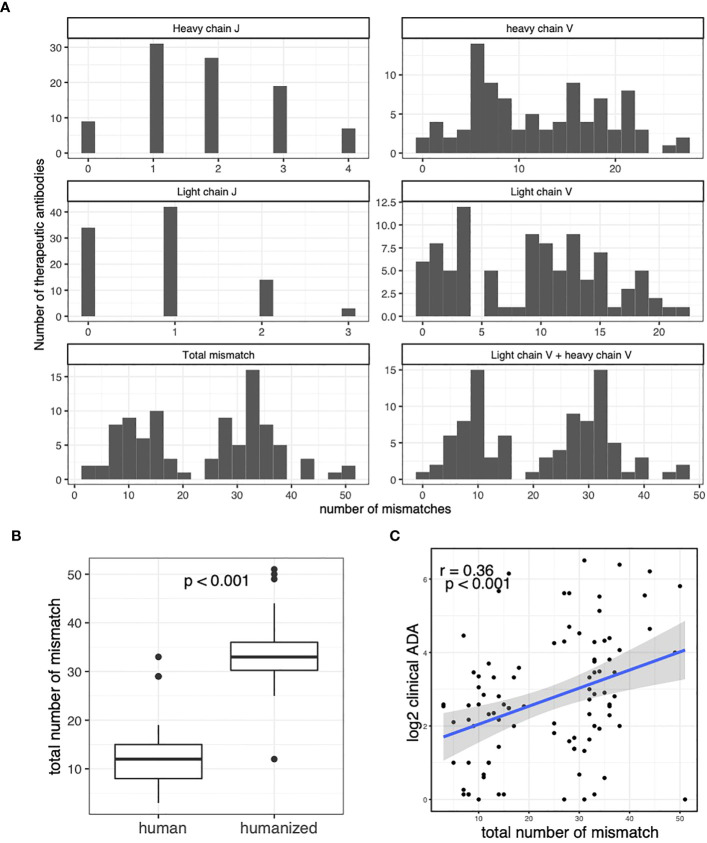
Caption: The clinical ADA rates are associated with the number of mismatches between therapeutic antibodies and the germline. **(A)** Histograms for number of mismatches between therapeutic antibodies and closest germline alleles. The number of mismatches in V and J regions of light and heavy chains are plotted. The total mismatch is the total number of mismatches in V and J regions of light and heavy chains. The “Light chain V + heavy chain V” shows the total number of mismatches in V regions of light and heavy chains. **(B)** The boxplot compares the number of total mismatches in human-origin antibodies and the humanized antibodies. **(C)** The Scatter plot shows the association between clinical ADA rate and the total number of mismatches in the therapeutic antibodies. T-test is used in **(B)** Spearman correlation is used to measure the associations in **(C)**.

We performed sequence alignment between antibody therapeutic sequence and somatic V/J alleles. We identified the closest somatic V and J alleles to each antibody therapeutic sequence. We found that the therapeutic antibodies use a diverse set of V and J alleles (see the accompanying dataset (32)). The sequence alignment also allows us to quantify the number of mismatches between the antibody therapeutic sequence and somatic V/J alleles (**Figure 2A**). Interestingly, we see a clear bi-modal distribution of the total number of mismatches, which is the sum of mismatches in heavy chain J gene, heavy chain V gene, light chain J gene and light chain V gene. Further investigation reveals that the bimodal distribution represents the difference between human-origin antibodies and the humanized antibodies (**Figure 2B**). The total number of mismatches are significantly correlated with the clinical ADA rate, suggesting that the introduced mutations increase the risk of immunogenicity of the therapeutic antibodies (**Figure 2C**).

### The immunogenicity of human-origin therapeutic antibodies are associated with V allele usage

**Figure 3:**
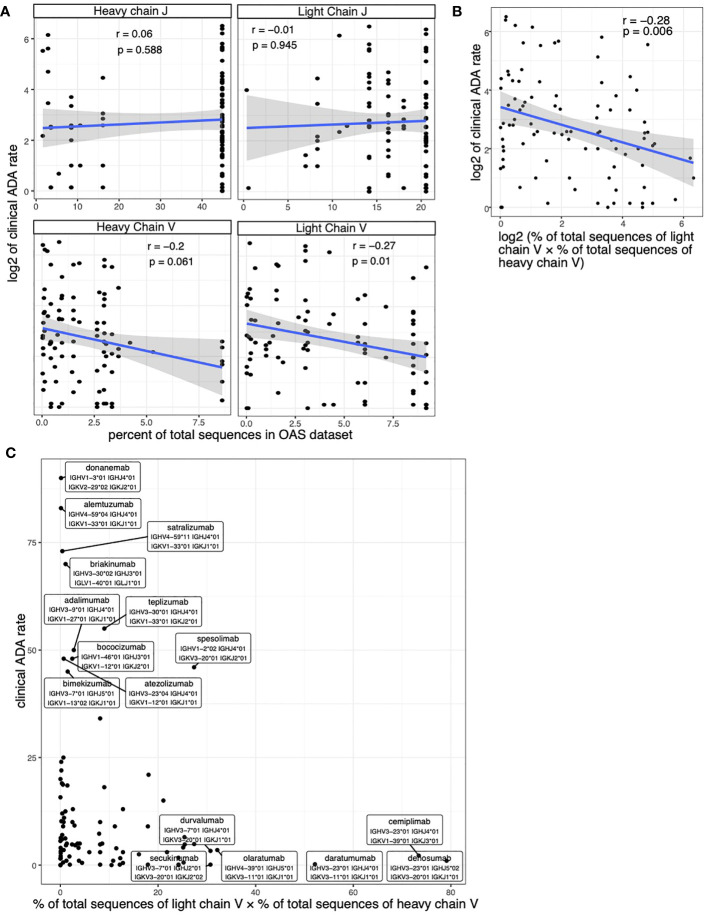
Assessing the correlation between clinical ADA rate and the allele usage of therapeutic antibodies. **(A)** Scatter plots show the association between clinical ADA rate and the usage of V and J alleles in light and heavy chains. **(B, C)** The scatter plot shows the association between clinical ADA rate and product of V allele usage in light and heavy chains. The log transformed data **(B)** and original data **(C)** is shown. Spearman correlation is used to measure the associations in **(A, B)**.

We examined the relationship between the usage of V/J alleles and the immunogenicity of the therapeutic antibodies. We found that the clinical ADA rate is positively correlated with the usage (measured as the percent of total sequences in OAS dataset) of V alleles in both light chain and heavy chains of the therapeutic antibody (**Figure 3A**). We did not see significant correlation between the ADA rate and J gene usage (**Figure 3A**).

We used the product of V allele usages in light chain and heavy chain to quantify the overall usage of V alleles of a therapeutic antibody. We again see a significant correlation between the V gene usage and the clinical ADA (**Figure 3B**). Most antibodies that have high clinical ADA rates use rare V alleles (alleles with low percent of total sequences in OAS dataset), such as donanemab and alemtuzumab (**Figure 3C**) The antibodies that used popular V alleles (alleles with high percent of total sequences in OAS dataset) tend to have low clinical ADA rate, such as cemiplimab, denosumab and daratumumab (**Figure 3C**).

The humanized antibodies have a higher number of mutations than the human-origin antibodies (**Figure 2B**). The higher number of mutations could affect our analysis in two ways. First, the number of mutations is directly correlated with ADA (**Figure 2C**). Second, the higher number of mutations makes it more difficult to identify the originating somatic V alleles used by the therapeutic antibody. Therefore, we next examined the relationship between ADA rate and V gene usage separately in humanized and human-origin antibodies.

**Figure 4:**
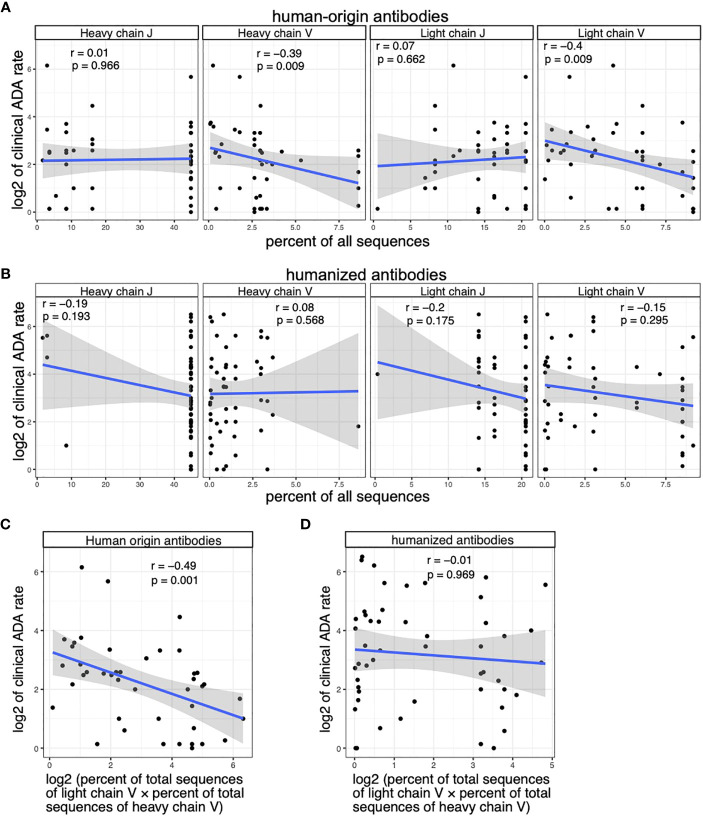
The clinical ADA is associated with the V allele usage of human-origin antibodies but not the V allele usage of humanized antibodies. **(A)** Scatter plots show the association between clinical ADA rate and the usage of V and J alleles in light and heavy chains of human-origin antibodies. Allele usage is quantified as the percent of total sequences with the allele in the OAS dataset. **(B)** Scatter plots show the association between clinical ADA rate and the usage of V and J alleles in light and heavy chains of humanized antibodies. Allele usage is quantified as the percent of total sequences with the allele in the OAS dataset. **(C)** The scatter plot shows the association between clinical ADA rate and product of V allele usage in light and heavy chains of the human-origin antibodies. **(D)** The scatter plot shows the association between clinical ADA rate and product of V allele usage in light and heavy chains of the humanized antibodies. Spearman correlation is used to measure the associations in **(A–D)**.

Among human-origin antibodies, the usages of V alleles in light chain and heavy chain, as well as their product, are significantly correlated with clinical ADA (**Figures 4A, C**). We did not see significant correlation between the ADA rate and J gene usage (**Figure 4A**). Among humanized antibodies, we did not see significant correlation between V/J usage and the clinical ADA (**Figures 4B, D**).

We further performed multivariable regression analysis to test the association between ADA rate and V gene usage among human-origin antibodies while controlling for other factors that could impact the immunogenicity, including the class of drug target, the disease category (based on drug’s first approved indication) and the year of drug’s first approval. The result of the multivariable regression again shows a significant association between V gene usage and clinical ADA rate (p value = 0.0038).

To summarize, our analysis showed that V alleles are associated with ADA in human-origin antibodies, but not in humanized antibodies. J alleles are not associated with ADA of human-origin and humanized antibodies.

### The immunogenicity of human-origin therapeutic antibodies is associated with the genomic variations of the V genes

**Figure 5:**
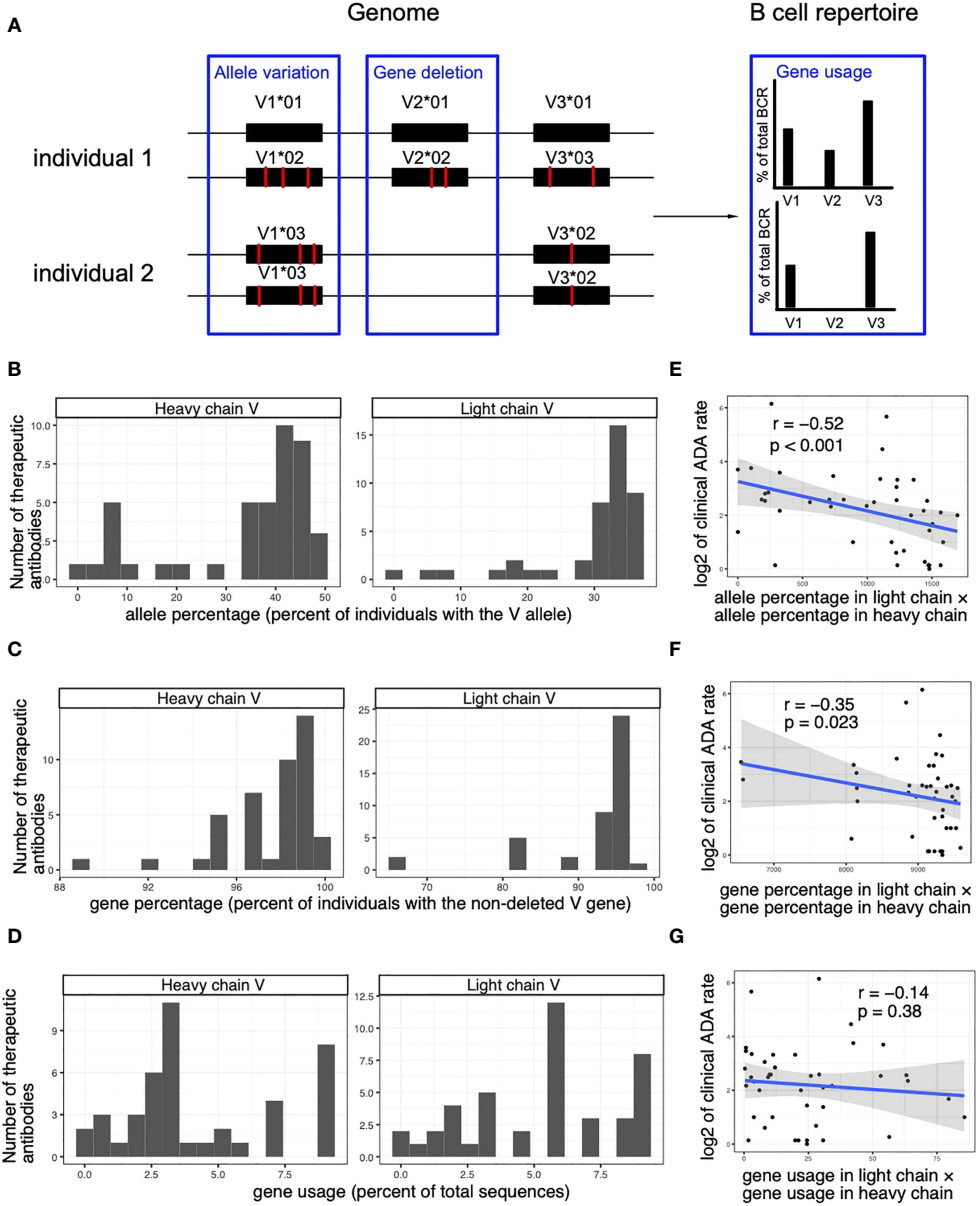
The immunogenicity of human-origin therapeutic antibodies is associated with the genomic variations of the V genes. **(A)** A schematic diagram demonstrating factors that affect the V allele usage. **(B)** Histograms show the frequencies of the V alleles (percent of individuals with the genomic V allele in OAS dataset) used in the heavy and light chain of the human-origin antibody drugs. **(C)** Histograms show the frequencies of non-deleted V genes (percent of individuals with the V gene in OAS dataset) of the V alleles used by the heavy and light chain of the human-origin antibody drugs. **(D)** Histograms show the usage of V genes (percent of total sequences with the V gene in OAS dataset) of the V alleles used by the heavy and light chain of the human-origin antibody drugs. **(E)** A scatter plot shows the correlation between clinical ADA rate and the percentage of V alleles (measured as the percent of individuals that carries the V allele in OAS dataset). The product of percentages in light chain and heavy chain is used to quantify the overall percentage of V alleles of a therapeutic antibody. **(F)** A scatter plot shows the correlation between clinical ADA rate and the percentage of non-deleted V genes (measured as the percent of individuals who carry the V gene OAS dataset). The product of percentages in light chain and heavy chain is used to quantify the overall percentage of V alleles of a therapeutic antibody. **(G)** A scatter plot shows the correlation between clinical ADA rate and the usage of V genes (measured as the percent of total sequences with the V gene in OAS dataset). The product of percentages in light chain and heavy chain is used to quantify the overall percentage of V alleles of a therapeutic antibody. Spearman correlations are used to measure the associations in **(D–F)**.

The usage of V alleles in a patient population is determined by both its availability in the genomes and the gene-level usage in the BCR repertoires. Specifically, the usage of V alleles can be decomposed into three factors: allele variation (30), gene deletion (26) and gene usage (28, 29) (**Figure 5A**). The deletion of V genes refers to the phenomenon that an entire V gene is missing in the genome of some individuals. For example, the IGHV2-70D gene is missing in 57% of individuals and IGHV4-30-2 gene is missing in 17% of individuals (26).

We used the OAS dataset to estimate the usage of the different V genes in BCR repertoires, the frequency of specific alleles of each V gene, and the frequency of deletions of the V genes (**Figures 5B–D**, see Methods for details). We next explored how the three factors contribute to the association between ADA rate and V allele usage. We found that the allele frequency and the gene deletion are significantly associated with the ADA rate of therapeutic antibodies (**Figures 5E, F**). In contrast, ADA is not significantly associated with the usage of V genes (**Figure 5G**). The results suggest that the genomic variations (allelic variation and gene deletion) in the population contribute to the immunogenicity of the therapeutic antibodies.

## Discussion

Therapeutic antibodies are a class of protein-based therapeutics that can elicit unwanted immune responses in patients (4). ADAs have been reported to affect the pharmacokinetics, efficacy and/or safety of the therapeutic antibodies (5–7, 9, 10). Therefore, a key goal of antibody engineering is to lower the risk of immunogenicity of antibody drugs. Currently, antibody engineering projects usually start with a group of candidates that have desirable affinity to the target molecule. Additional mutagenesis are then used to further improve the binding affinity. The immunogenicity risk assessment usually happens after the affinity optimization. Multiple methods are used to assess the immunogenicity risk, including in silico methods (MHC-II binding prediction and “humanness” assessment), *in vitro* methods (DC loading assay, MAPPS assay and T cell activation assays) and *in vivo* animal models (rat and non-human primates) (33–40). Molecules with high immunogenicity risk may require additional modifications to reduce the potential immunogenicity. However, the additional modifications could impact affinity, leading to another round of costly cycle of antibody engineering. In addition, the preclinical immunogenicity risk assessments may not fully predict the ADA rate in clinical trials. Therefore, it is critical to reduce the immunogenicity risk of therapeutic antibodies from the beginning of an antibody engineering project.

The result of our study shows that the usage of V alleles is associated with the ADA rate of the human-origin therapeutic antibodies. The results suggest that antibody engineering efforts should start with antibody frameworks that use popular v alleles to reduce the risk of immunogenicity. Our analysis also identified a significant correlation between immunogenicity and the number of mismatches between the antibody sequence and somatic sequences. The result suggests that mismatch number should be considered as a factor in prioritizing antibody candidates during the drug development process.

Our analysis shows that the V allele usage is correlated with the immunogenicity of human-origin antibodies, but not with the immunogenicity of humanized antibodies. This may be due to the fact that there is a large number of mismatches between humanized antibodies and the somatic sequence. Because the V chain has been heavily modified, the usage of the originating V chain may not play a major role in the immunogenicity of the humanized antibody. In addition, the mismatch makes it difficult to reliably identify the originating V chain of the humanized antibody. In fact, a portion of the humanized antibodies are derived from animals, especially in the CDR regions. Furthermore, some of the humanized antibodies used consensus sequences of multiple human V genes rather than any individual human V gene (41, 42). These antibodies do not have a single originating V gene from humans. Taken together, it is not surprising that the V gene usage is not correlated with the immunogenicity of the humanized antibodies.

The study is an observational study. Further studies are needed to establish the causal relationship between V allele usage and immunogenicity. However, existing understanding on the mechanism of immunogenicity suggests that the V allele usage contributes to the immunogenicity. Due to the variation between alleles, antibodies that use rare V alleles are more likely to be seen as foreign antigen in patients that do not carry the allele. The foreign sequence could serve as both T cell and B cell epitope that leads to the development of ADA.

The current study analyzed the immunogenicity data at the drug level. In future studies, the patient-level data from clinical trials will be valuable to further understand the relationship between V gene usage and immunogenicity. If there is a causal relationship between V allele variation and immunogenicity, we expect the ADA risk to be lower in patients who carry the same allele used by the antibody drug and higher in patients who don’t carry the allele. Results from the patient level study will help us better understand the personalized ADA risk of patients.

## Data availability statement

The datasets presented in this study can be found in online repositories. The names of the repository/repositories and accession number(s) can be found below: https://figshare.com/articles/dataset/Mab_VJ_datasets/22796135.

## Author contributions

Conceptualization: ZH and SS. Methodology: ZH. Data collection: ZH and SC. Data analysis: ZH. Writing – Original Draft Preparation: ZH. Writing – Review & Editing: ZH, SS and SC. Visualization: ZH. Supervision: SS. All authors contributed to the article and approved the submitted version.
